# A reconstruction of global hydroclimate and dynamical variables over the Common Era

**DOI:** 10.1038/sdata.2018.86

**Published:** 2018-05-22

**Authors:** Nathan J. Steiger, Jason E. Smerdon, Edward R. Cook, Benjamin I. Cook

**Affiliations:** 1 Lamont-Doherty Earth Observatory of Columbia University, Palisades, NY 10964, USA; 2 NASA Goddard Institute for Space Studies, New York, NY 10025, USA

**Keywords:** Hydrology, Natural hazards, Palaeoclimate, History, Hydrology

## Abstract

Hydroclimate extremes critically affect human and natural systems, but there remain many unanswered questions about their causes and how to interpret their dynamics in the past and in climate change projections. These uncertainties are due, in part, to the lack of long-term, spatially resolved hydroclimate reconstructions and information on the underlying physical drivers for many regions. Here we present the first global reconstructions of hydroclimate and associated climate dynamical variables over the past two thousand years. We use a data assimilation approach tailored to reconstruct hydroclimate that optimally combines 2,978 paleoclimate proxy-data time series with the physical constraints of an atmosphere—ocean climate model. The global reconstructions are annually or seasonally resolved and include two spatiotemporal drought indices, near-surface air temperature, an index of North Atlantic variability, the location of the intertropical convergence zone, and monthly Niño indices. This database, called the Paleo Hydrodynamics Data Assimilation product (PHYDA), will provide a critical new platform for investigating the causes of past climate variability and extremes, while informing interpretations of future hydroclimate projections.

## Background & Summary

Hydroclimate extremes, including persistent droughts and pluvials, can have extensive effects on societies and ecosystems. For example, multi-year droughts in California have caused significant agricultural losses, tree mortality, forest fires, and other impacts (e.g., refs 1–4). While the frequency of hydroclimate extremes are estimated to increase with global warming (e.g., refs 5–8), it is unclear if the underlying climate dynamics of such events are accurately produced in climate model simulations (e.g., refs 9–11) and if the models capture the full range of extremes on decadal and centennial time scales^12,13^. The lack of clarity on these issues is due in part to the lack of global reconstructions of droughts along with their associated dynamics. For example, the instrumental era lacks multidecadal ‘megadroughts’, known to have occurred throughout the past two millennia^14,15^. Hydroclimate reconstructions that include dynamical information therefore provide critical long-term perspectives and allow for better characterizations of the risks associated with future hydroclimate variability.

This work uses a data assimilation (DA) method to derive the first global reconstructions of hydroclimate and associated dynamical variables that span the last two millennia. In contrast to traditional reconstruction approaches and existing paleo-hydroclimate products, DA-based reconstruction methods can simultaneously estimate both hydroclimate fields and corresponding atmosphere-ocean states^16^; having these two components is critical for analyses of the causes of hydroclimate extremes. DA for paleoclimate works by optimally fusing proxy information with the dynamical constraints of climate models^17–21^. Here we use proxy information from a network of 2,978 annually resolved proxy-data time series together with the Community Earth System Model Last Millennium Ensemble (CESM LME) of climate model simulations^22^. We perform three separate global reconstructions over the Common Era (the past 2000 years) at annual resolution (defined as April to the next calendar year March), the boreal growing season June through August (JJA), and the austral growing season December through February (DJF). We specifically reconstruct three global variables gridded at ~2 degree resolution: surface temperature at 2 m, the Palmer drought severity index (PDSI), and the standardized precipitation evapotranspiration index (SPEI). We also reconstruct the following climate indices at annual, JJA, and DJF temporal resolutions: the global mean temperature, the North Atlantic sea surface temperature index which is the non-detrended and non-smoothed version of the Atlantic multidecadal oscillation index (AMO), and the location of the intertropical convergence zone (ITCZ)^23^ in 11 longitudinal zones. Additionally we reconstruct the monthly Niño sea surface temperature (SST) indices (Niño 1+2, 3, 3.4, 4) and the monthly equatorial Pacific zonal SST gradient^24^. We reconstruct these specific climate index variables because they have been linked to hydroclimate variability and extremes across the globe. For example, Atlantic and tropical Pacific modes of variability have been linked to drought across North America (e.g, refs 25–32). Also, shifts in the ITCZ and consequent monsoonal changes over land have, for example, been associated with the collapse of the Maya Civilization^33,34^ and the demise of the Angkor Empire^35^.

This Paleo Hydrodynamics Data Assimilation product (PHYDA), represents the first collection of global hydroclimate reconstructions along with their associated dynamical variables. It is also the first DA-based paleoclimate reconstruction to explicitly include the location of the ITCZ and monthly SST indices. We have also made several innovations in the reconstruction methodology compared to previous approaches^19,20,36–38^, including an algorithm that is more general and an order of magnitude faster, bias correction of the climate model variables, improved proxy system modeling, and a greatly expanded proxy network. Here we provide a thorough validation of all the reconstructed variables and also include a robust uncertainty estimation for all variables. These reconstructions can be used to more clearly elucidate the dynamics associated with droughts and pluvials on time scales ranging from seasons to centuries over the past two millennia: for example, they can be useful for assessing the causes of droughts in equatorial Africa or multidecadal droughts in the American West^16^. Additionally, the reconstructions are relevant for assessing model simulations and can be used to evaluate model projections of future hydroclimate variability and change (cf. refs 39,40).

## Methods

### Data assimilation

We employ a DA technique that optimally combines proxy data or observations with climate model states. The model provides an initial, or prior, state estimate that is updated based on the proxy observations and an estimate of the errors in both the observations and the prior. The general state update equations of DA^41^ can be written as

(1)xa=xb+K[y−H(xb)],

where

(2)K=BHT[HBHT+R]−1 .

In these equations, **x**_*b*_ is the prior (or ‘background’) estimate of the state vector and **x**_*a*_ is the posterior (or ‘analysis’) state vector; the state vector contains all of the variables that are to be reconstructed. Observations (or proxies) are contained in **y**. The observations are estimated by the prior through $H(x_{b})$, which is, in general, a nonlinear vector-valued observation operator that maps **x**_*b*_ from the state space to the observation space. **B** is the prior covariance matrix, **R** is the error covariance matrix for the proxy data, and **H** represents a linearization of H. In a general sense, the reconstruction process works by computing an optimal linear fit between the initial guess of the climate state, the prior **x**_*b*_, and the proxies **y**. Because proxies are available at annual time steps, a reconstruction is made by iteratively computing equations (1) and (2) for each year (or season within each year) of the existing proxy data.

We implement the general DA equations described above by using an ensemble square root filter from ref. 42. Though the authors of ref. 42 recommend the sequential assimilation of observations for computational reasons, the simultaneous assimilation of observations is actually an order of magnitude faster in contemporary matrix-optimized computing software, such as MATLAB. We therefore modified previous approaches used for paleoclimate DA^19,20,36–38^ and specifically implemented the matrix equations from ref. 42, as listed below in equations (3), (4), (5), (6), (7), (8), (9). These equations begin with the prior ensemble state estimate **x**_*b*_, which is an *m*×*n* matrix where *m* is the state size (e.g., if only temperature fields are reconstructed then *m* will be the number of grid points in the spatial temperature field) and *n* is the ensemble size. The prior is then separated into an *m*×1 ensemble mean ${\bar{x}}_{b}$ and the *m*×*n* deviations from this mean ${x'}_{b}^{}$ (the mean is removed from each row of **x**_*b*_). The implementation subsequently updates the ensemble mean and the deviations from the ensemble mean separately:

(3)x¯a=x¯b+K[y−H(x¯b)] ,

(4)x'a=x'b−K˜H(x'b) ,

where the parantheses in $H({\bar{x}}_{b})$ and $H({x'}_{b}^{})$ denote the operator **H** acting on ${\bar{x}}_{b}$ and ${x'}_{b}^{}$. The observation vector **y** is of dimension *p*×1, where *p* is the number of proxy data values available in a given time interval (e.g., a year or season) and $H({\bar{x}}_{b})$ and $H({x'}_{b}^{})$ have respective dimensions of *p*×1 and *p*×*n*. The two Kalman gain matrices are calculated as

(5)K=BHT[HBHT+R]−1,

and

(6)K˜=BHT{[HBHT+R]−1}T[HBHT+R+R]−1 ,

where **R** is the *p*×*p* observational-error covariance matrix, square roots indicate a matrix square root, −1 superscripts indicate a matrix inverse, T superscripts indicate a matrix transpose and where

(7)BHT=1n−1x'b[H(x'b)]T,

and

(8)HBHT=1n−1H(x'b)[H(x'b)]T.

After computing equations (3), (4), (5), (6), (7), (8) the full posterior ensemble is then recovered through

(9)xa = x¯a+x'a ,

where ${\bar{x}}_{a}$ is added to each column of ${x'}_{a}^{}$. Collectively, equations (3), (4), (5), (6), (7), (8), (9) are computed for each year (or a particular season of each year) to arrive at a series of posterior ensemble state estimates that together constitute the probabilistic spatiotemporal reconstruction. In the reconstruction files for all variables (Data Citation 1) we have included the posterior ensemble mean, 1 standard deviation of the posterior ensemble as well as its 5th, 50th, and 95th percentiles; this error estimate explicitly includes uncertainty information from the spread in the climate model prior (**HBH**^**T**^) as well as the error in the proxy models (**R**).

In our implementation of equations (3), (4), (5), (6), (7), (8), (9), **R** is assumed to be a diagonal covariance matrix (uncorrelated errors) where the entries are the error variance of each proxy (defined in the ‘Proxy system models’ section). If the proxy errors are correlated then equations (3), (4), (5), (6), (7), (8), (9) can be computed in the same way using a non-diagonal **R**.

### Climate model data and reconstruction variables

As in previous studies^19,20,36–38^ we use an offline DA approach in which **x**_*b*_ is the same for each year and is drawn from an existing climate model simulation: the ensemble members are seasonally or annually-averaged climate states instead of an ensemble of independently running model simulations, as in traditional online DA. This approach therefore propogates no information forward in time (e.g., **x**_*a*_ from year *t*−1 is not used as **x**_*b*_ in year *t*) and only the proxies constrain the time evolution of the reconstruction. In principle, the ensemble members can be drawn from a single long simulation, multiple simulations or even from simulations of a collection of climate models; to be informative for the reconstruction, the prior should be representative of what one is trying to reconstruct (e.g., to reconstruct a year with a large volcanic eruption, the prior should include ensemble members that contain such events). According to many previous reconstruction experiments^19,20,36–38,43^ year-specific forcing or boundary condition information appears to be unnecessary for skillful reconstructions as long as the prior is sufficiently representative. Furthermore, the offline approach can be performed without the immense computational costs of a traditional online approach.

We construct the prior **x**_*b*_ using the CESM LME^22^, which used atmosphere and land components with ~2-degree resolution and ocean and sea ice components with ~ 1-degree resolution. The simulations were run from the years 850 to 1850 CE using estimates of the transient evolution of solar intensity, volcanic emissions, greenhouse gases, aerosols, land-use conditions, and orbital parameters^44^. The simulations were given identical forcings but differed by round-off error in the initial atmospheric state; this difference was sufficient to generate simulations with different internal ocean-atmosphere variability and therefore different time histories (e.g., annual Niño 3.4 indices from the simulations are uncorrelated). For the reconstruction prior, we used a single simulation, number 10 from the full-forcing ensemble, to generate our prior ensemble; specifically, we used the middle 998 years of the CESM simulation excluding the two simulation endpoints to create a static 998 member prior ensemble that was used to estimate the climate state in each year of the reconstruction (the last year cannot be used because of the particular annual averaging we used here and the first year cannot be used because the variable SPEI integrates the previous 12 months of climate information, therefore only the second year of an SPEI time series is meaningful). This prior is consistent with previous work that has established that the prior is not required to contain year-specific forcing or boundary condition information, rather it must merely be statistically representative of the reconstruction period^19,20,36–38,43^. Here and in theoretical tests of the methodology^38^ we performed sensitivity tests with different members from the CESM LME and found no discernible differences in the results. Previous work has shown that the choice of climate model prior has little impact on the DA reconstructions when the choice is among the publicly available millennial-length coupled simulations^20^. However, model biases in the temperature and precipitation fields are specific to each model and can influence the fidelity of the reconstructions. To partially account for issues related to biases, we bias-corrected the climate model temperature and precipitation fields by replacing their monthly mean climatologies with observational monthly mean climatologies from refs 45,46.

Reconstructions were performed from the years 1–2000 CE targeting three different temporal windows: annual means (defined as April to the next calendar year March), the boreal growing season of JJA, and the austral growing season of DJF. The particular annual average used herein was chosen to account for the seasonal cycle of a global network of proxies as well as climate phenomena like the El Niño—Southern Oscillation, the continuity of which would be ignored with a calendar year average. Except for the monthly Niño SST indices described below, all other variables were reconstructed over the annual, JJA, or DJF windows.

Each reconstruction contains the following gridded fields over the global domain: 2 m air temperature, PDSI, and SPEI using a 12-month decaying exponential weighting kernel^47^ chosen to closely resemble the time scale of PDSI; the potential for skillful reconstruction of these fields was previously demonstrated using pseudoproxy experiments^16^. Both PDSI and SPEI were computed using the Penman-Monteith equation for potential evapotranspiration and monthly climate model output of precipitation, 2 m temperature, vapor pressure, net surface radiation, surface pressure, and surface wind (estimated from 10 m down to 2 m using the wind profile power law); the climatologically bias-corrected temperature and precipitation fields were used in the calculations. PDSI was computed using the MATLAB code from ref. 48, which produces the standard formulation of PDSI as opposed to self-calibrating versions (e.g., ref. 49). Both PDSI and SPEI are broadly used in drought monitoring^50^ and historical drought reconstructions^14,15,51–54^.

We also reconstruct the following index variables: the area-weighted global mean temperature, the North Atlantic SST index which is the non-detrended and non-smoothed version of the Atlantic multidecadal oscillation (AMO), the monthly Niño SST indices (Niño 1+2, 3, 3.4, 4), the monthly equatorial Pacific zonal SST gradient^24^, and the location of the intertropical convergence zone (ITCZ) in 11 longitudinal zones. Because there are different smoothing and standardization conventions in computing Niño SST indices, we have simply computed the area-averaged monthly SST values in each Niño region. We use the definition of the location of the ITCZ from^23^, which is the expected value of precipitation (*P*) using a 10^th^-power area weighting, integrated over the tropical latitudes *ϕ*_1_ and *ϕ*_2_,

(10)ϕITCZ=∫ϕ1ϕ2ϕ[cos(ϕ)P]10dϕ∫ϕ1ϕ2[cos(ϕ)P]10dϕ

In equation (10), we use the annual or seasonally averaged precipitation and also *ϕ*_1_=30^°^S and *ϕ*_2_=30^°^N to account for Monsoon regions where the seasonal precipitation maximum can extend far beyond the equator. Following refs. 23,55 we interpolate the tropical precipitation to a 0.1 degree latitudinal grid before computing equation (10). The 11 longitudinal zones are listed in the tables and include all major ocean and land regions in the tropics (e.g., continental Africa and the Atlantic) and for different definitions of these regions (e.g., different definitions of the Pacific ITCZ sector). Note that for computation simplicity, all of the reconstructed index variables are included in the prior state vector rather than being post-processed from reconstructed spatial climate fields. The monthly indices are reconstructed by the appended state method where here for example, each monthly index occupies 12 elements in the state vector of a given ensemble member.

### Proxy data

**Figure 1:**
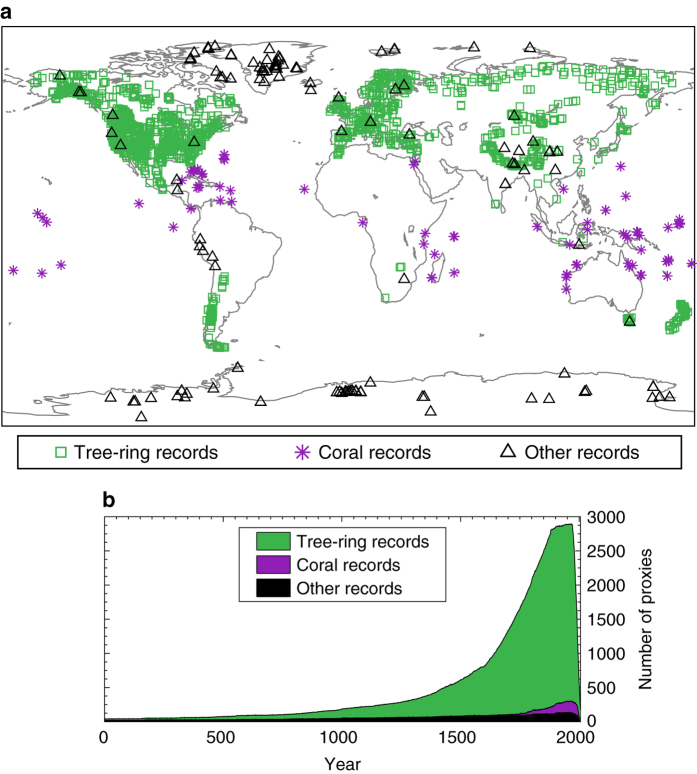
Proxy data network and temporal availability. **(a)** Spatial distribution of the combined proxy data network from refs. 56 and 57. Proxies are categorized by how they are modeled in the proxy system modeling framework: 2591 tree rings, 197 corals and sclerosponges, and 190 other records, which include 153 ice core isotope records, 26 speleothem isotope records, 10 lake sediment records, and 1 marine sediment record. **(b)** Temporal availability of the proxy network by proxy type.

Two proxy databases form the foundation of the database: the updated PAGES2k database^56^ and the tree-ring width collection of ref. 57. Additionally, 59 publicly available proxy records including ice cores, speleothems, and lake sediments were also included. In total this database includes 2,978 annually resolved proxies after removing duplicates; only annually or seasonally resolved proxy data values are used such that we include only the annually resolved portions of mixed-resolution proxies. This constitutes the largest multiproxy database employed thus far in a global reconstruction. Figure 1 shows the spatial and temporal distribution of the combined proxy network, with the numbers of each proxy type indicated in the caption. Note that the ice core, speleothem, and sediment records have been grouped together because they are modeled similarly in the proxy system modeling framework (see the following section). Age model uncertainties for the relevant proxy types are only accounted for through our use of the best estimate of the annual ages as determined by the authors of each proxy dataset. The full proxy database and additional proxy metadata is publicly available (Data Citation 2).

### Proxy system models

DA-based reconstructions must use climate model variables to estimate proxy observations ($H(x_{b})$ in Equation 1). For example, a given climate model's temperature and precipitation can be used to estimate tree-ring width through a sub-model. Such ‘forward models’ are referred to in paleoclimatology as proxy system models (PSMs)^58^. Here we employ statistical, regression-based PSMs that are specific to each proxy; this improves on previous DA-based reconstructions that used only a univariate linear regression with temperature for all proxies^20^. We first illustrate this procedure for the ‘other records’ in Fig. 1. The PSMs for these proxies are derived from linear regressions between the *i*^th^ annual proxy time series, *p*_*i*_, and the local instrumental temperature series from ref. 45 indicated by *X*_*i*_. Each *p*_*i*_ is standardized to unit variance and for the three different reconstructions discussed previously, *X*_*i*_ is either an annual, JJA, or DJF average. The regression equation is

(11)pi=αi+βiXi+εi,

which is calculated over the available temporal overlap between the proxy and the instrumental time series within the calibration period 1920–2000 (leaving approximately 5 decades for a verification interval of 1871–1919, consistent with many previous studies, e.g., refs 59–61). We chose to throw out all proxies that did not have at least 20 overlapping values for the regression (for all proxy types this amounted to a total of 82 proxies that were not used because they did not extend sufficiently through the calibration interval). The prior estimate of the proxies, $H{\left(x_{b}\right)}_{i}$, is then found for each proxy by using the calibrated parameters *α*_*i*_ and *β*_*i*_ in

(12)H(xb)i=αi+βiXi ,

where *X*_*i*_ are the corresponding climate model temperature grid point values nearest to the proxy location in each prior ensemble member. The vector of residuals for each proxy, *ε*_*i*_, are then used to compute the diagonal entries of the matrix **R**, where the *i*th entry is computed as $R_{i}=\overset{¯}{\varepsilon_{i}^{2}}$. Note that this statistical model only considers local information and relies solely on the Kalman gain covariance relationships to inform non-local climate variables. We did not employ a physically-based PSM for the oxygen isotope proxies, such as for ice-core *δ*^18^O, because of the lack of available high resolution millennial-length simulations with water istopes and because previous work has indicated no improved reconstruction performance using such PSMs over a linear regression with local temperature^38^.

The PSM for tree rings is modeled similar to the approach above but with either local instrumental temperature or local instrumentally-derived PDSI^62^, depending on which instrumental data type has the highest absolute correlation with the proxy over the calibration period. This correlation is computed using the averaging time scale of the reconstruction such that it is possible for a given site to be modeled with temperature for one time average and PDSI for another time average. For the annual reconstruction, 1719 tree-ring chronologies were modeled with PDSI while 872 were modeled with temperature; for the JJA reconstruction 1579 were modeled with PDSI and 1012 with temperature; and for the DJF reconstruction 1572 were modeled with PDSI and 1019 with temperature. Using both temperature and PDSI in the PSMs is necessary because of the heterogenous sensitivities of different tree-ring sites and the inclusion of both tree ring-width and density; additionally, using both temperature and moisture sensitive trees is essential for producing a skillful DA-based reconstruction of both temperature and moisture fields^16^.

We employ a bivariate regression-based PSM based on ref. 63 for the coral and sclerosponge *δ*^18^O proxies. This PSM uses both SST and sea surface salinity to estimate proxy *δ*^18^O. Here we calculate regression parameters for each site individually using the long-term ocean reanalysis from ref. 64 instead of basin or region-wide parameter values as used in ref. 63. For non-*δ*^18^O coral proxies, we employ a linear univariate regression with SST.

### Code availability

The MATLAB code (https://www.mathworks.com/products/matlab.html) necessary to perform the reconstructions discussed in this data descriptor are available at https://github.com/njsteiger/PHYDA under a free BSD license. The reconstructions were performed using MATLAB version R2015a.

## Data Records

Each of the three reconstructions constituting the first version of PHYDA are publicly available at the Zenodo data repository as NetCDF4 files (Data Citation 1), which include all of the reconstructed variables and their uncertainties; specifically this includes the posterior ensemble mean, 1 standard deviation of the posterior ensemble as well as its 5th, 50th, and 95th percentiles. The NetCDF4 format also incorporates all of the associated variable metadata. The paleoclimate proxy database used herein is also publicly available at the Zenodo data repository (Data Citation 2).

## Technical Validation

We validate the reconstructions against observations primarily using two skill metrics: Pearsons' correlation (r) and the mean continuous ranked probability skill score (CRPSS). Correlation is computed using only the reconstruction mean time series at each grid point while the CRPSS metric accounts for both the mean grid point time series as well as the grid point uncertainty estimates. CRPSS is based on the continuous ranked probability score (CRPS), which is a ‘strictly proper’ scoring rule that accounts for the skill of the entire posterior reconstruction distribution^65^. CRPS penalizes bias, incorrect variance, incorrect phasing, and an ensemble spread that is either too wide or overconfident. Because the posterior ensemble estimates are approximately normally distributed we use equation (5) from ref. 66,

(13)crps=σ{yn[2Φ(yn)−1]+2ϕ(yn)−1π},

where *y*_*n*_=(*y*−*μ*)/*σ*, with *y* being the observed value, *μ* the mean of the posterior ensemble estimate, and *σ* the standard deviation of the posterior ensemble, and where *ϕ*(*y*_*n*_) and Φ(*y*_*n*_) are respectively the normal probability density function and the normal cumulative distribution function of *y*_*n*_. Note that this implementation assumes that there is no error in the observations. All of our uses of equation (13) are for time series, either individual time series or grid point time series. We therefore compute the mean of all the time-step values of equation (13) and denote it as CRPS. The skill score version, CRPSS, is the reconstructed CRPS computed with respect to the CRPS of a reference distribution, CRPSS≡1−CRPS_rec_/CRPS_ref_, here the initial uninformed prior. We use CRPSS instead of CRPS because CRPS has the referenceless range of [0, ∞) while CRPSS has the range (−∞, 1] with positive CRPSS indicating that the reconstructed distribution is more skillful for this metric than the uninformed prior. CRPSS is generally a more stringent skill metric than correlation, so we focus here primarily on CRPSS. Additionally, for validating the time series reconstructions we use the metrics of the coefficient of efficiency^67^ and the cross-spectral coherence computed using a multi-taper method^68^.

**Figure 2:**
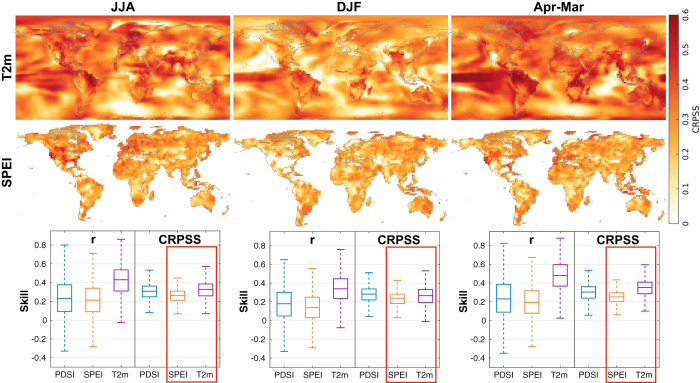
Skill assessments for the temperature and hydroclimate reconstruction products computed over the years 1901–2000. The **top row** shows the mean continuous ranked probability skill score (CRPSS) for the 2 m temperature reconstruction (T2m). CRPSS is computed for each grid point time series with the observational temperature dataset of Berkeley Earth^45^. The **middle row** shows the CRPSS for the standardized precipitation-evapotranspiration index (SPEI). The observational SPEI is computed with a 12-month decaying exponential kernel and using the CRU TS3.23 land surface datasets^69^. The **bottom row** summarizes the spatial skill values from the top two rows (orange and purple CRPSS box plots contained within the red boxes) as well as corresponding CRPSS assessments for the Palmer drought severity index (PDSI) and also correlation (r) skill values over the same time period. For the PDSI assessments, we use the observational product of ref. 62.

The top two rows of Fig. 2 show the skill of the reconstructed 2 m temperature and SPEI at each grid point using the CRPSS skill metric. The bottom row of Fig. 2 summarizes the spatial skill in box plots for all the spatial variables using r in addition to CRPSS. Seasonal (JJA and DJF) and annual reconstructions are organized by column. The skill metrics are computed for the years 1901–2000 against Berkeley Earth^45^ for temperature and an observational SPEI computed with a 12-month decaying exponential kernel and using the CRU TS3.23 land surface datasets^69^; the interval of 1901–2000 is chosen because CRU TS3.23 only extends back to the year 1901. The skill assessments do not include Antarctica because of the sparsity of observational data in this region and because hydroclimate indices are not suited for use over ice-covered landscapes. Assessments of PDSI are included in the bottom row of Fig. 2. We note that the reconstruction uses standard PDSI while the observational verification data^62^ uses the slightly different self-calibrating version of PDSI.

It is important to note that in Fig. 2 we compute the skill metrics over the interval 1901–2000 while the parameters of the PSMs are fit to observations over the interval of 1920–2000. In traditional reconstruction techniques (e.g., ref. 70) it would not be suitable to show validation statistics over the calibration interval because the instrumental data are used to both fit the proxy data and also for the reconstruction target. In contrast, the PSM parameter fitting here is not an equivalent process because the target field is a pre-industrial climate model simulation and the temporal information is only derived from the proxies. However, in validating these reconstructions we do not rely solely on skill metrics computed over a significant fraction of the PSM calibration interval. In Tables 1, 2, 3 we additionally compute skill metrics for the temperature-based climate indices over the period 1871–1919.

**Figure 3:**
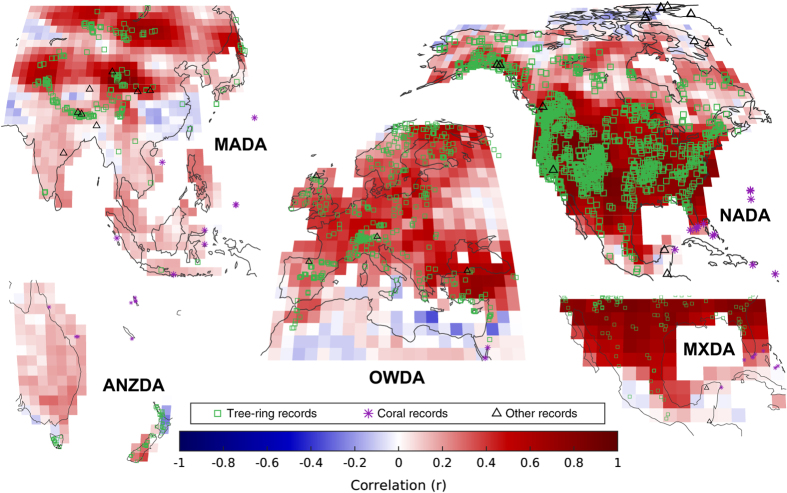
Reconstruction assessment using drought atlases. Correlation between the data assimilation-based PDSI reconstruction and the PDSI reconstructions of the drought atlases from the years 1500–2000: the North American drought atlas (NADA)^14,51^, the Old World drought atlas (OWDA)^15^, the Monsoon Asia drought atlas (MADA)^52^, the Mexican drought atlas (MXDA)^53^, and the Australia and New Zealand drought atlas (ANZDA)^54^. Correlations are computed using the JJA reconstruction for NADA, OWDA, MADA, and MXDA and the DJF reconstruction for ANZDA. Prior to computing the correlations, the drought atlases were interpolated to the land surface grid of the CESM LME model simulation used in the data assimilation-based reconstruction presented here.

Skill tends to be highest in the tropics and nearby the proxy locations (cf. Fig. 1) during the summer growing season, as is evident, for instance when comparing JJA and DJF reconstruction skill over North America. The annual results also compare favorably with the seasonal reconstructions, particularly over the tropics, showing that it is possible to provide skillful results across a range of time intervals using this DA approach, thus verifying the theoretical results of previous pseudoproxy experiments^16^. The box plots in the bottom row of Fig. 2 show generally consistent results across the seasons and variables, though the temperature reconstructions are generally more skillful than the PDSI or SPEI reconstructions, while PDSI and SPEI are fairly comparable. Though not shown, the spatial patterns of r are similar to those of CRPSS: for example, the JJA SPEI spatial correlation between the r and CRPSS maps is 0.70 while the corresponding spatial correlation between r and CRPSS for JJA 2 m temperature is 0.68. However, unlike CRPSS, r is consistently high across regions that possess many proxies; this can be seen, for example, when contrasting the CRPSS metric of JJA SPEI (Fig. 2) with r of JJA PDSI in northern Mexico (Fig. 3).

As further validation, we also compare the PDSI reconstructions to the available Drought Atlases^14,15,51–54^ (Fig. 3). Each of the drought atlases have been extensively validated and represent the current gold-standard PDSI reconstruction product. The correlations in Fig. 3 cover the period 1500–2000 and indicate a DA reconstruction that is strongly consistent with the drought atlas products, particularly over North America and Europe where in some locales correlations approach 1; similarly high correlations exist for 100 year intervals through time as well as for the entire length of each drought atlas (which have heterogeneous start times). The agreement between the reconstructions and the drought atlases is remarkable given the vastly different methods used to derive the PDSI fields and that the proxy datasets were not designed to contain the same inputs nor use the same proxy data processing methods (though there is some overlap between the proxy network used herein and the proxy networks of the drought atlases). However, we note a more muted agreement for the ANZDA and also in regions where the proxy network used in the DA reconstruction has limited or no data; where there is little or no data, the prior ensemble often cannot be sufficiently constrained, resulting in localized regions of low skill^16,19,38^. The two skill metrics presented in both Fig. 3 and Fig. 2 provide complimentary measures for establishing the skill of the spatial field reconstructions.

**Figure 4:**
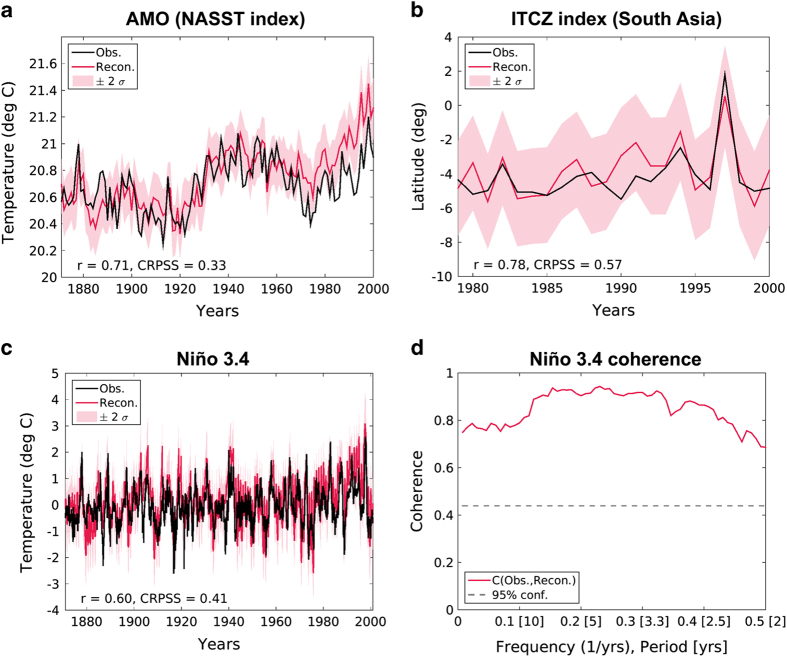
Reconstruction verification for three representative climate-dynamical indices. **(a)** Verification of the annual mean North Atlantic sea surface temperature index (NASST), which is the non-detrended, non-smoothed version of the Atlantic multidecadal oscilation (AMO). This panel includes the AMO observations (Obs.) ^76^ and the mean reconstruction (Recon.) with a corresponding ±2*σ* range of the posterior ensemble. Skill values are indicated for correlation (r) and the mean continuous ranked probability skill score (CRPSS) in the bottom left corner. These skill values are computed over the entire time interval shown. **(b)** Reconstruction verification of the location of the annual mean intertropical convergence zone (ITCZ) over the South Asian monsoon region spanning the tropics from 65^°^E to 95^°^E. The observational ITCZ is computed using the Global Precipitation Climatology Project version 2.3 (ref. 46) available back to the year 1979. Skill values of r and CRPSS are computed over the time interval shown here. **(c)** Reconstruction verfication for the monthly Niño 3.4 index, similar to (a) and (b). The observational Niño 3.4 index is computed from Berekley Earth surface temperature dataset^45^, which over the ocean is based on HadSST^77^. **(d)** Coherence as a function of frequency and period between the mean Niño 3.4 reconstruction and the observations shown in panel (**c**).

The reconstructed dynamical climate indices span the globe and include many drivers of hydroclimate variability. Figure 4 shows representative verifications of three of these indices: (a) the AMO, (b) the location of the ITCZ over the South Asian monsoon region, and (c) the monthly Niño 3.4 index, with Fig. 4(d) showing the cross-spectral coherence of the reconstructed and observation-based Niño 3.4 index. The panels in Fig. 4 illustrate that a range of important climate indices from different regions are skillfully reconstructed, with high positive correlations, CRPSS, and coherence values. We highlight in particular that these are the first DA-based paleoclimate reconstructions of the location of the ITCZ and monthly Niño indices.

We have additionally performed an exhaustive verification of all the reconstructed index variables. Tables 1, 2, 3 present several skill metrics for each of the temperature-based variables: r and CRPSS over the interval 1871–2000, r and CRPSS over the interval 1871–1919, cross-spectral coherence at the specific periods of 2.5, 5, and 10 years (as in Fig. 4 where the full range of coherence is shown), and CE using the verification mean of 1871–1919 (mimicking a traditional calibration—validation skill test, where here the calibration period is the period over which the PSMs were trained). We note, however, that unlike CRPSS, CE is not a strictly proper scoring metric for ensemble reconstructions^65^; we also note that both r and CRPSS do not incorporate two time periods as in CE, so r (or r^2^) is not directly comparable with CE in the manner traditionally used in statistical dendroclimatology^71^. These skill values are all shown for the annual (Table 1), JJA (Table 2), and DJF (Table 3) reconstructions. Many variables show skill (positive values) across many or all metrics with some variables having particularly high values, such as global mean temperature (GMT) with r=0.88, CRPSS=0.56, and CE=0.77 (Table 1). A few variables, such as the monthly Niño 1+2 index, appear to have skill only at multiyear time scales, low r and negative CRPSS and CE yet high coherence at 2.5, 5, and 10 year periods (Table 1). We note that the negative CE values for the monthly Niño indices (Tables 1, 2, 3) are the result of an annual cycle that shows up too strongly in the reconstructions; r does not account for variance, coherence is looking at multiyear time scales where there is not a variance issue, and CRPSS considers several factors in the reconstruction that outweigh too much variance in this instance. At an annual average of the indices, when the annual cycle is averaged out, all CE values improve; for the reconstruction using annual PSMs and using the tropical annual average defined previously, Niño 1+2 CE=0.37, Niño 3 CE=0.34, Niño 3.4 CE=0.35, Niño 4 CE=0.07, and ΔSST Pacific CE=0.12 (cf. the corresponding column in Table 1). Tables 4, 5, 6 show the r and CRPSS metrics for all of the ITCZ reconstructions, which are limited to the period of 1979–2000 because the Global Precipitation Climatology Project version 2.3 (ref. 46) is only available back to the year 1979. All of the ITCZ reconstructions are skillful in at least one season, though the skill in some regions is strongly dependent on the season, e.g., the Tropical East Africa annual mean versus JJA and DJF (top row in Tables 4, 5, 6).

The series of validation tests presented in this section include 2 measures of spatial skill, r and CRPSS, 18 box plots summarizing the spatial skill of all spatially-resolved variables, and 6 tables with a total of 168 entries verifying the skill of the reconstructed climate indices. These assessments have been done with different skill metrics over three different time intervals (both observational and paleo time intervals) to ensure that a robust picture of each variable's skill can be seen. These validation tests show that many variables are skillfully reconstructed but the level of skill is dependent on the region, the variable, the season (e.g., JJA versus DJF), and the timescale (e.g., annual versus decadal). Future versions of PHYDA will include high-resolution climate model simulations for the prior from the upcoming Paleoclimate Model Intercomparison Project phase 4 (ref. 72), including a more sophisticated bias-correction scheme (e.g., refs 73,74), updates to the proxy network (such as the inclusion of all the tree-ring records used in the drought atlases), and updates to the PSMs as they become further developed.

## Usage Notes

Paleoclimate reconstructions rely on a relatively sparse network of noisy proxy data time series and the reconstruction may have significant uncertainty depending on the variable, the location, and the time period of interest^75^. Before using PHYDA for analyses, users should consult the relevant spatial verification Figs. 2,3 or Tables (1, 2, 3, 4, 5, 6) to determine whether the variables of interest can provide useful information. It is also important to consider the range of uncertainty on the variable of interest (included in the NetCDF4 files) and not just the ensemble mean. Because of the decreasing proxy availability further back in time (Figure 1b) the uncertainty in the reconstruction correspondingly increases. Because of how the DA reconstruction methodology is formulated, decreasing amounts of information from proxies will yield a corresponding decrease in the variance of the ensemble mean reconstruction as the prior becomes more heavily relied upon; this gradual reduction in variance of the ensemble mean should not be interpreted as a reduction in the variance of the historical climate.

## Additional information

**How to cite this article**: Steiger, N. J. *et al.* A reconstruction of global hydroclimate and dynamical variables over the Common Era. *Sci. Data* 5:180086 doi: 10.1086/sdata.2018.86 (2018).

**Publisher**’**s note**: Springer Nature remains neutral with regard to jurisdictional claims in published maps and institutional affiliations.

## Supplementary Material



## Figures and Tables

**Table 1:** Verification of annual mean index variables and monthly SST variables.

Variable	r (1871–2000)	CRPSS (1871–2000)	r (1871–1919)	CRPSS (1871–1919)
GMT	0.88	0.56	0.35	0.41
AMO	0.71	0.33	0.48	0.53
Nino 1+2	0.18	-0.12	0.18	−0.10
Nino 3	0.45	0.24	0.42	0.28
Nino 3.4	0.60	0.41	0.55	0.45
Nino 4	0.59	0.44	0.37	0.37
ΔSST Pacific	0.10	-0.01	0.11	0.04
	coh 2.5 yr (1871–2000)	coh 5 yr (1871–2000)	coh 10 yr (1871–2000)	CE (1871–1919)
GMT	0.42	0.53	0.74	0.77
AMO	0.40	0.38	0.61	0.39
Nino 1+2	0.70	0.81	0.56	−2.69
Nino 3	0.83	0.87	0.70	−1.11
Nino 3.4	0.86	0.90	0.79	−0.16
Nino 4	0.87	0.87	0.74	−0.17
ΔSST Pacific	0.17	0.67	0.45	−2.88
Verifications include the area-weighted global mean temperature (GMT), the North Atlantic SST index which is the non-detrended and non-smoothed version of the Atlantic multidecadal oscillation (AMO), the monthly Niño SST indices (Niño 1+2, 3, 3.4, 4), and the monthly equatorial Pacific zonal SST gradient (ΔSST Pacific). Skill metrics include correlation (r), mean continuous ranked probability skill score (CRPSS), coherence at the specific periods of 2.5, 5, and 10 years, and the coefficient of efficiency (CE). Each metric includes the period over which it was computed except for CE where the years indicate the verification mean time period.				

**Table 2:** Verification of JJA mean index variables (GMT and AMO) and monthly SST variables (Niño 1+2, 3, 3.4, 4 and ΔSST Pacific), cf. Table 1.

Variable	r (1871–2000)	CRPSS (1871–2000)	r (1871–1919)	CRPSS (1871–1919)
GMT	0.87	0.60	0.51	0.42
AMO	0.72	0.42	0.47	0.42
Nino 1+2	0.19	−0.10	0.22	−0.06
Nino 3	0.45	0.20	0.48	0.24
Nino 3.4	0.56	0.32	0.56	0.29
Nino 4	0.50	0.31	0.39	0.19
ΔSST Pacific	0.07	0.01	0.06	0.07
	coh 2.5 yr (1871–2000)	coh 5 yr (1871–2000)	coh 10 yr (1871–2000)	CE (1871–1919)
GMT	0.56	0.59	0.70	0.80
AMO	0.38	0.48	0.53	0.49
Nino 1+2	0.69	0.83	0.46	−2.65
Nino 3	0.81	0.88	0.63	−1.32
Nino 3.4	0.85	0.91	0.71	−0.55
Nino 4	0.87	0.86	0.62	−0.79
ΔSST Pacific	0.13	0.41	0.29	−2.85

**Table 3:** Verification of DJF mean index variables (GMT and AMO) and monthly SST variables (Niño 1+2, 3, 3.4, 4 and ΔSST Pacific), cf. Table 1.

Variable	r (1871–2000)	CRPSS (1871–2000)	r (1871–1919)	CRPSS (1871–1919)
GMT	0.79	0.34	0.20	0.33
AMO	0.55	0.13	0.09	0.28
Nino 1+2	0.19	−0.19	0.18	−0.21
Nino 3	0.44	0.10	0.40	0.15
Nino 3.4	0.55	0.23	0.51	0.34
Nino 4	0.59	0.27	0.44	0.37
ΔSST Pacific	0.10	−0.06	0.13	−0.06
	coh 2.5 yr (1871–2000)	coh 5 yr (1871–2000)	coh 10 yr (1871–2000)	CE (1871–1919)
GMT	0.58	0.41	0.66	0.42
AMO	0.17	0.52	0.37	−0.11
Nino 1+2	0.60	0.78	0.58	−3.09
Nino 3	0.75	0.88	0.78	−1.89
Nino 3.4	0.75	0.88	0.87	−0.93
Nino 4	0.79	0.87	0.82	−0.92
ΔSST Pacific	0.31	0.77	0.54	−3.19

**Table 4:** Verification of the ITCZ variables at annual resolution.

Region	Longitudinal range (deg E)	r (1979–2000)	CRPSS (1979–2000)
Tropical East Africa	[28, 50]	0.59	0.32
Indian Ocean	[50, 95]	0.71	0.52
South Asia	[65, 95]	0.78	0.57
Indonesia	[95, 130]	0.72	0.33
East Pacific Ocean	[130, 170]	−0.01	0.13
Pacific Ocean	[130, 260]	0.77	0.41
Pacific Ocean	[160, 260]	0.80	0.37
West Pacific Ocean	[170, 260]	0.83	0.36
Tropical South America	[260, 320]	0.19	0.16
Atlantic Ocean	[320, 345]	0.35	0.35
Tropical Africa	[345, 50]	−0.05	0.26
Regions are named and given the longitudinal ranges that they cover (in degrees eastward from the Prime Meridian, in keeping with the convention of climate model grids.) Each metric includes the period over which it was computed.			

**Table 5:** Verification of the ITCZ variables at JJA mean resolution, cf. Table 4.

Region	Longitudinal range (deg E)	r (1979–2000)	CRPSS (1979–2000)
Tropical East Africa	[28, 50]	−0.04	0.30
Indian Ocean	[50, 95]	0.64	0.52
South Asia	[65, 95]	0.36	0.31
Indonesia	[95, 130]	0.41	0.33
East Pacific Ocean	[130, 170]	0.05	0.15
Pacific Ocean	[130, 260]	0.71	0.48
Pacific Ocean	[160, 260]	0.73	0.49
West Pacific Ocean	[170, 260]	0.75	0.49
Tropical South America	[260, 320]	0.29	0.27
Atlantic Ocean	[320, 345]	−0.08	0.27
Tropical Africa	[345, 50]	−0.33	0.22

**Table 6:** Verification of the ITCZ variables at DJF mean resolution, cf. Table 4.

Region	Longitudinal range (deg E)	r (1979–2000)	CRPSS (1979–2000)
Tropical East Africa	[28, 50]	0.04	0.27
Indian Ocean	[50, 95]	0.33	−0.13
South Asia	[65, 95]	0.32	−0.01
Indonesia	[95, 130]	0.40	0.27
East Pacific Ocean	[130, 170]	0.20	0.12
Pacific Ocean	[130, 260]	0.32	0.32
Pacific Ocean	[160, 260]	0.34	0.27
West Pacific Ocean	[170, 260]	0.40	0.28
Tropical South America	[260, 320]	0.57	0.34
Atlantic Ocean	[320, 345]	−0.09	0.10
Tropical Africa	[345, 50]	0.33	0.04
